# Ce(III) and La(III) ions adsorption using Amberlite XAD-7 resin impregnated with DEHPA extractant: response surface methodology, isotherm and kinetic study

**DOI:** 10.1038/s41598-023-37136-7

**Published:** 2023-06-20

**Authors:** Azadeh Yarahmadi, Mohammad Hassan Khani, Masoud Nasiri Zarandi, Younes amini, Ali Yadollahi

**Affiliations:** 1 Department of Chemical, Petroleum and Gas Engineering, Semnan University, Semnan, Iran; 2 Nuclear Fuel Cycle Research School, Nuclear Science and Technology Research Institute, P.O.BOX 11365-8486, Tehran, Iran

**Keywords:** Chemistry, Engineering

## Abstract

In this paper, the removal efficiency of Cerium (Ce(ΙΙΙ)) and lanthanum (La(ΙΙΙ)) ions from aqueous solution using Amberlite XAD-7 resin impregnated with DEHPA(XAD7-DEHPA) was studied in the batch system. The adsorbent ( XAD7–DEHPA) was characterized by SEM–EDX, FTIR and BET analysis Techniques. The response surface methodology based on the central composite design was applied to model and optimize the removal process and evaluate operating parameters like adsorbent dose (0.05–0.065), initial pH (2–6) and temperature (15–55). Variance analysis showed that the adsorbent dose, pH and temperature were the most effective parameters in the adsorption of Ce(ΙIΙ)and La(IΙI) respectively. The results showed that the optimum adsorption condition was achieved at pH = 6, the optimum amount of absorbent and the equilibrium time equal to 0.6 gr and 180 min, respectively. According to the results, the adsorption percentage of Ce(ΙIΙ) and La(ΙΙΙ) ions onto the aforementioned resin were 99.99% and 78.76% respectively. Langmuir, Freundlich, Temkin and sips isotherm models were applied to describe the equilibrium data. From the results, Langmuir isotherm (R^2^ (Ce) = 0.999, R^2^ (La) = 0.998) was found to better correlate the experimental rate data. The maximum adsorption capacity of the adsorbent ( XAD7–DEHPA) for both Ce(IΙI) and La(III) was found to be 8.28 and 5.52 mg g^−1^ respectively. The kinetic data were fitted to pseudo-first-order, pseudo-second-order and Intra particle diffusion models. Based on the results, the pseudo-first-order model and Intra particle diffusion model described the experimental data as well. In general, the results showed that ( XAD7–DEHPA) resin is an effective adsorbent for the removal of Ce(IΙI) and La(III) ions from aqueous solutions due to its high ability to selectively remove these metals as well as its reusability.

## Introduction

The unique and functional properties of rare earth elements (REEs) in various industries have caused these elements to be highly regarded in recent decades and referred to as industrial vitamins^1,2^. Lanthanum and cerium are used as raw materials for high-tech industries. The applications of these elements in metallurgy, agriculture, chemicals, ceramics petrochemical, opticals, electronics and nuclear technologies are diverse and the growth of demand for these materials in international markets has led to an increase in the prices of rare earth elements in recent years^3–5^. With the increase in demand for rare earth elements, the balance between the world demand and the natural affluence of the REEs in ores, often created as the balance problem, is a serious issue for REEs preparator^6–8^.

These elements are found in nature in combination and in combination with each other, and their separation due to very similar physical and chemical properties requires many costly processes. Therefore the separation and recovery of these elements from secondary sources have become very important^9,10^. To recover and remove ions of rare earth elements from aqueous solution and effluents different methods such as solvent extraction, chromatography, crystallization Partial, ion exchange, and adsorption are discussed^11,12^. Many of these methods are non-economic, expensive, low efficiency to remove ions, and combined with production They are secondary contaminants and are useless for low concentrations. Selecting the best methods and materials for wastewater treatment, it is very important that, the factors such as efficiency, cost and environmental issues must be considered^13^. In recent decades, due to the applications of solvent-impregnated resins in the removal and separation of metal ions, the use of this method has been developed. The basic principle in this application method includes ion exchange and solvent extraction^14^. Solvent-impregnated resins (SIRs) have the advantages of ion exchange resins and solvent extraction, but there are no environmental complexities and problems due to solvent extraction in this method^15^.

In SIRs, the chelating reagent sits on the resin with great power and acts like an ion exchanger. However, in some cases, the mechanism of solvent extraction by SIRs is very similar to solvent extraction. During impregnation and SIR preparation, the extraction was homogeneously dispersed into the porous tissue of the resin. The extraction used must be able to act as a solvent extraction method and yet show a strong tendency for the resin to adsorb ions^16^.

Due to the lack of movement of the extraction in the pores of the resin, more amounts of it are retained in the resin. Other advantages of this method include good mechanical stability, high surface area and reusing possibility^17^.

In general, various studies have been performed on the separation and removal of rare earth elements using different methods. Archana Kumari et al.^18^ investigated the extraction of rare earth metals from chloride medium by organometallic complexation using di-2-Ethylhexyl phosphoric acid (D2EHPA) and the best separation factors for Nd/La is 17.73 at pH 2 and for Pr/La is 12.69 at pH 1.7 were obtained. The sorption of rare earth elements was investigated using bone powder by Monica Butnariu et al.^19^. Studies have shown that the maximum sorption of these elements occurs in PH = 4 and the equilibrium time is ˂ 240 min. Sorption data were also better described by Langmuir isotherms compared to Redlich-Peterson and Freundlich isotherms.

Measuring kinetic of extraction of Sm(III) by the single drop technique from nitrate solution using bis(2,4,4-trimethylpentyl) dithiophosphinic acid (Cyanex 301) and 2-Ethylhexyl phosphoric acid (D2EHPA). Torkaman et al.^20^ showed that the Cyanex 301 extractant provided a slower extraction rate than that of the D2EHPA extractant, therefore, the extraction rate of Sm(III) with D2EHPA was better than that of Cyanex 301. Furthermore, they concluded that the extraction process is mainly controlled by the diffusion mechanism.

Zhuo Chen et al.^21^ have studied The extraction process of Nd(III), Eu(III) and Er(III) using membrane dispersion micro-extractors, The residence time and the dispersed droplets’ diameter determine of aqueous phase flow rate, The findings of this study showed that As the aqueous phase flow rate increases, the efficiency first decreases and then increases.

Torkaman et al.^22^ investigated the solvent extraction of gadolinium (III) by Cyanex301, D2EHPA and a combination of extractants from an aqueous nitrate solution, The results showed that the best extraction efficiency was done at pH 4, the concentration of DEHPA 0.06 M and and concentration of Cyanex301 0.5 M. Moreover, they reported that a mixture of extractants performed better for gadolinium extraction than their single system.

Removal studies of trivalent samarium using activated biochar derived from cactus fibers from Aqueous Solutions were investigated. Various parameters affecting initial metal concentration, contact time, pH, ionic strength and temperature on samarium adsorption were investigated. Maximum samarium uptake was noted to be 350 g/kg, under pH = 6.5 (Loukia Hadjittofi et al.)^23^.

Devlina Das et al.^24^ have studied the Recovery of La(III) from an aqueous solution with biosorbents of plant and animal origin in the batch and column systems. According to the results, the maximum adsorption capacity of La(III) under optimum conditions (biomass dose: 0.3, 0.2 g/L. time: 4, 3 h. pH: 6. initial metal concentration: 300 mg/L. temperature:50 °C) of fish scales and neem sawdust onto the aforementioned biosorbents were 200.0 mg/g and 160.2 mg/g, respectively.

In recent years, many statistical and mathematical methods have been used to design effective factors in chemical processes. Response surface methodology (RSM) is one of the statistical and mathematical methods for designing and optimizing processes. This method can have the effects of parameters showing the independent and relative importance of the interaction between two or more variables in the process. The RSM method not only specifies the optimal conditions but also suggests a suitable regression model, using different designs such as central composite design (CCD) or box-Behnken design (BBD)^25,26^.

The objective of the present paper is to investigate the performance of Amberlite XAD-7 resin impregnated with DEHPA to remove Ce(III) and La(III) from an aqueous solution in the batch adsorption process. This work focused on the modification of the Amberlite XAD-7 surface with a simple chelating agent named DEHPA. This novel sorbent is subsequently applied for heavy metal adsorption namely Ce(III) and La(III).

Also, individual and simultaneous effects of process variables, environment pH, adsorbent dose and temperature are discussed in the adsorption process using the RSM based on the CCD at five levels. This paper also compares Ce(III) and La(III) adsorption equilibrium data with the Langmuir, Freundlich, Temkin and Sips isotherms and compares the Ce(III) and La(III) adsorption kinetic data with the pseudo-first-order, pseudo-second-order and Intra particle diffusion models.

## Experimental

### Materials

Amberlite XAD-7 resin was a non-ionic resin with an acrylic ester structure produced by Fluka. Dimensions of the prepared resin are 297 to 841 microns, its dry and wet densities are equal to 1.24 and 1.05 g/cm^3^ respectively, its surface area is 450 m^2^/g and its average pore volume is 1.14 cm^3^/g.

DEHPA (di-2-ethylhexyl) phosphoric acid with a molar mass of 322.43 g/mol and a density of 98 was supplied by the German company Merck. D2EHPA extraction has acidic properties as an organic extractant and extracts at a low pH value.

Lanthanum nitrate (La(NO_3_)_3_.6H_2_O), cerium nitrate (Ce(NO_3_)_3_.H_2_O) salts for the solution of metal ions, sodium hydroxide (NaOH), nitric acid (HNO_3_) used to adjust the pH of the solution were all made by Merck. Organic solvent toluene (C_6_H_5_CH_3_) with 99.99% purity was prepared from Merck company and used as a diluent. All chemicals used in the present study were of analytical grade and were used without further purification.

### Instrumentation and characterization of SIR

In this study, an Italian-made Gallenkamp mechanical shaker was used to stir the solutions. To measure the solution concentration of Ce(IΙI) and La(ΙIΙ) ions, the inductively coupled plasma-atomic emission spectrometry (ICP-AES) device model AXTurbo150 of Varian company was used at wavelengths of 379.478 and 413.464 nm, respectively. To determine the pH of the solution, a pH meter of the Swiss model 780 was used.

Characterization of the impregnated resins (SIRs) using the fourier transform infrared (FTIR), PerkinElmer Spectrum, 10.30.06, USA, between 400 and 4000 cm^−1^ wavenumber was obtained. For the determination of surface area and porosity were used scanning electron microscopy (SEM), VEGA//SEM, Tescan, A.S, Czech Republic, was integrated with Scanning Electronic Microscope- Energy Dispersive X-ray (EDX), Rontech, German.

### Impregnation procedure

**Figure 1:**
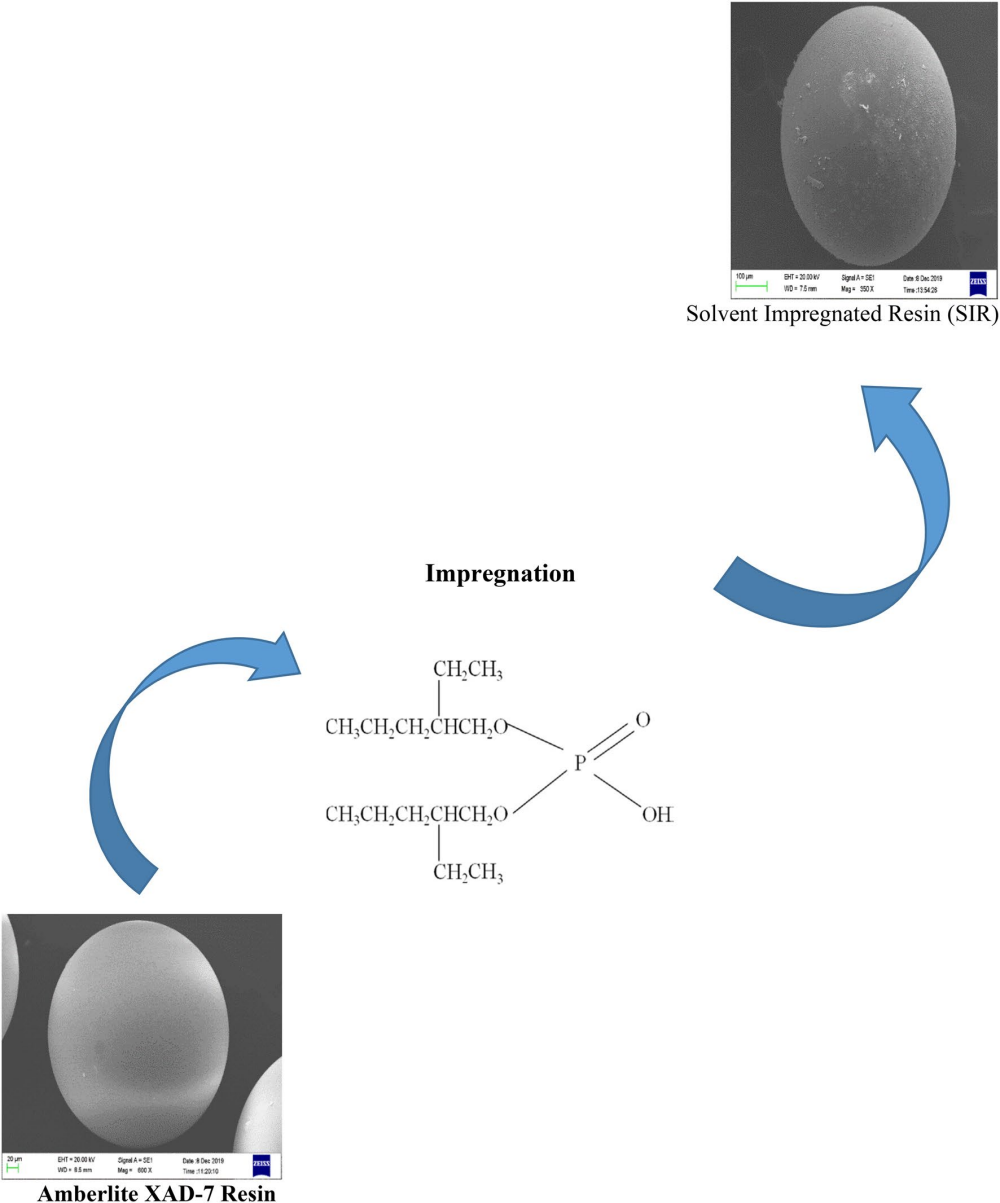
The schematic of the impregnation process of Amberlite XAD-7 resin with a solvent.

To impregnate Amberlite XAD-7 resin with DEHPA, the first 20 g of resin was washed three times with double distilled water to remove its impurities and prepare the internal pores of the resin for the impregnation process with the extraction. The washed resin was then passed through a filter and dried completely at room temperature for 48 h. Then, 15 g of dry resin with 30 ml of DEHPA was contacted at 0.5 M in toluene at room temperature on a stirrer at a constant speed for two hours. The resin grains were then separated from the organic phase and washed several times with 2 M nitric acid (HNO_3_) to make the sub-filter solution free of the organic phase. Finally, the resin grains were washed with distilled water and dried completely at room temperature for 48 h. The schematic of the impregnation process of Amberlite XAD-7 resin with solvent is shown in Fig. 1.

### Central composite design

Examining the impact of independent variables on the process increases the number of experiments, time and cost. Response surface methodology is a set of effective and practical methods for accurate and economical evaluation of variables and process response and can perform process optimization in addition to modeling. In the response surface method, it is possible to obtain large amounts of data with fewer tests. In this method, in addition to evaluating the main and interaction effects of different parameters, the optimal value of each parameter can be calculated. The surface response method can be done using different designs such as Box–Behnken design, face-centered cube design (FCCD) and central composite design. The CCD is a quadratic application design in the RSM that uses 5 levels for each independent variable, consisting of a central point and two factorial points that are ± 1 unit away from the center point. There are also two-star points that make it possible to estimate the curvature and are far from the center point ±$\propto$^27–29^.

To predict response behavior, a quadratic polynomial equation is defined according to Eq. 1.

1 $$\mathrm{Y}=\upbeta_{0}+\sum_{i=0}^{K}{\beta }_{ii}{X}_{i}+\sum_{\mathrm{i}=0}^{\mathrm{K}}{\beta }_{ii}{X}_{i}^{2}+\sum_{i,j=0}^{K}{\beta }_{ij}{X}_{i}{X}_{j}$$

where Y is the predicted answer, $\upbeta_{0}$ is constant coefficients, $\upbeta_{\mathrm{i}}$ is the linear coefficient, $\upbeta_{\mathrm{ii}}$ is quadratic coefficient and $\upbeta_{\mathrm{ij}}$ is the interaction coefficient also K is the number of independent variables and xi and xj demonstrate the independent variable. Also, the rate of change of the variable per unit is denoted by Δx. In Eq. 2, x_i_ is the encoded value of i variable the actual value. The variable is represented by X_i_ and the actual value of the variable is represented by x_0_ at the central point^30–32^.

2 $${\mathrm{x}}_{\mathrm{i}}=\frac{{\mathrm{x}}_{i}-{\mathrm{x}}_{0 }}{\Delta \mathrm{x}}$$

### Batch adsorption

The adsorption of Ce(ΙIΙ) and La(ΙΙI) ions in the batch system was investigated as a function of pH, contact time, initial concentration of metal ion solution and process temperature. For adsorption experiments, the first 20 ml of a solution containing Ce(ΙIΙ) and La(ΙΙI) ions with a certain concentration was transferred to 100 ml polyethylene containers and its pH was adjusted using nitric acid (HNO_3_) and sodium hydroxide (NaOH) 0.1 M. Then a certain amount of resin to Excess solution and polyethylene containers were placed in a mechanical shaker for a specified period of time at a specified temperature. After the desired time, the resins were separated from the solution using filter paper and the equilibrium concentrations of Ce(ΙIΙ) and La(ΙΙI) ions in the solution were measured using an inductively coupled plasma-atomic emission spectrometry (ICP-AES). All experiments were repeated twice.

The amount of adsorbed metal ions per unit mass of resin (resin adsorption capacity for metal ions (mg g^−1^) was calculated using the following Eq. (3):

3 $$q=({c}_{i}-{c}_{e})\times \frac{V}{M}$$

And the percentage of metal ion adsorption was calculated by the following Eq. (4):

4 $$\% \,\mathrm{Adsorption}= \frac{{c}_{i}-{c}_{e}}{ci}\times 100$$

Here, C_i_ and C_e_ are the initial and equilibrium concentrations (mg L^−1^), respectively. V is the volume of the solution (L) and M is the mass of the dry adsorbent (g)^33^.

### Desorption studies

The ability to recover the adsorbent for several adsorption and desorption cycles is one of its most important characteristics for the economic evaluation of the adsorption process^34^.

After adsorption tests to reuse the used resins, they were washed with nitric acid (HNO_3_) at concentrations of 0.1 and 2 to release the lanthanum and cerium metals and reusable. To do this, the used resins are contacted with 10 ml of nitric acid (HNO_3_) with the mentioned concentrations for 24 h in a shaker at 150 rpm, and then the resin is separated using filter paper and rinsed with distilled water. Then placed at room temperature to dry and be reusable.

## Results and discussion

### Characteristics of DEHPA-impregnated XAD-7 resin

#### FTIR

**Figure 2:**
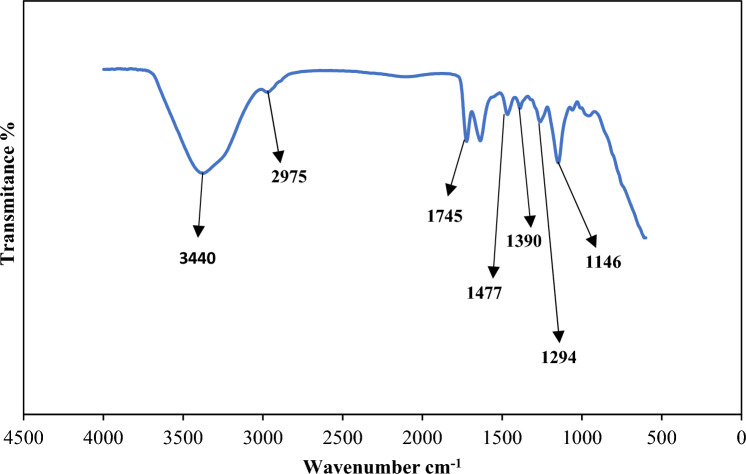
FTIR spectra of the Amberlite XAD-7.

**Figure 3:**
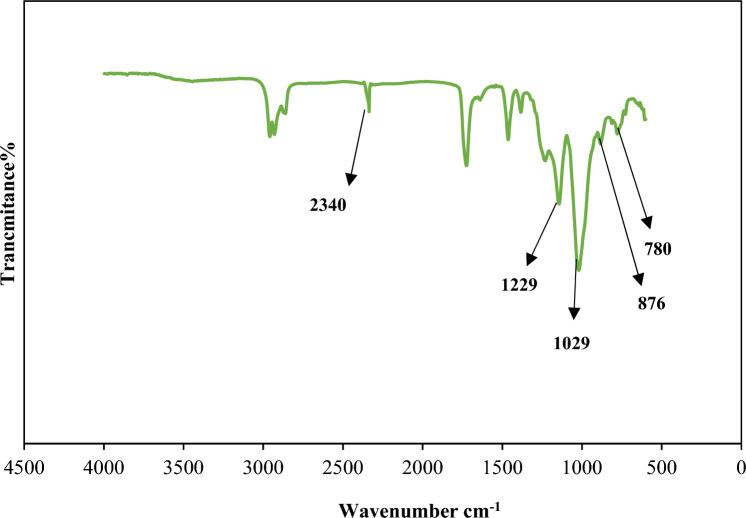
FTIR spectra of the Amberlite XAD-7 resin impregnated with DEHPA (XAD-DEHPA).

**Figure 4:**
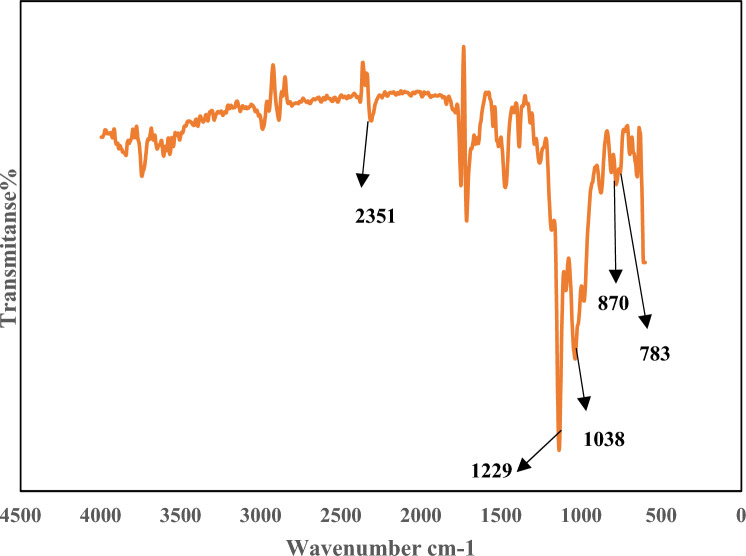
FTIR spectra of the Amberlite XAD-7 resin impregnated with DEHPA (XAD-DEHPA) after adsorption of Ce(III) and La(III).

Figures 2, 3 and 4 indicate the FT-IR adsorption frequencies and the assignments of the specific bands of the Amberlite XAD-7, XAD7-DEHPA and XAD7-DEHPA after adsorption of La(IΙI) and Ce(ΙIΙ), respectively.

From the spectrum of XAD‒7 (Fig. 2), the aliphatic C–H group stretching CH_3_ group can be seen at 2975 cm^−1^ and 1477 cm^−1^. The bond at 1745 cm^−1^ is due to the C=O stretching frequency. The peak at 1390 cm^−1^ can be assigned to the C-H deformation of CH_3_. As can be seen from the figure, C–O stretching in ester group at 1146 and 1294 cm^−1^. Moreover, the wide sharp peak at 3440 cm^−1^ corresponds to O–H^9^.

In the spectra of XAD7-DEHPA (Fig. 3), the bands at 780, 876 and 1029 cm^−1^ are assigned to P–C–O stretching. The presence of two bodies located at 1229 and 2340 cm^−1^ is indicated by P = O and P–OH stretching. The mentioned bands are due to the presence of an organophosphorus acid-specific group on Amberlite XAD-7 and are proof of the success of the process of impregnation of Amberlite XAD-7 using DEHPA extraction^16,35^.

The FTIR spectrum of XAD7-DEHPA after adsorption of La and Ce in Fig. 4 shows the -wavelength of some XAD7-DEHPA peaks has changed after the adsorption process. Also, multiple new peaks at 600–800, 1500–1800 and 3000–4000 cm^−1^ regions appear which may be due to the adsorption of Ce(IΙI) and La(ΙIΙ) ions on Amberlite XAD-7 resin impregnated with DEHPA.

#### SEM–EDX

**Figure 5:**
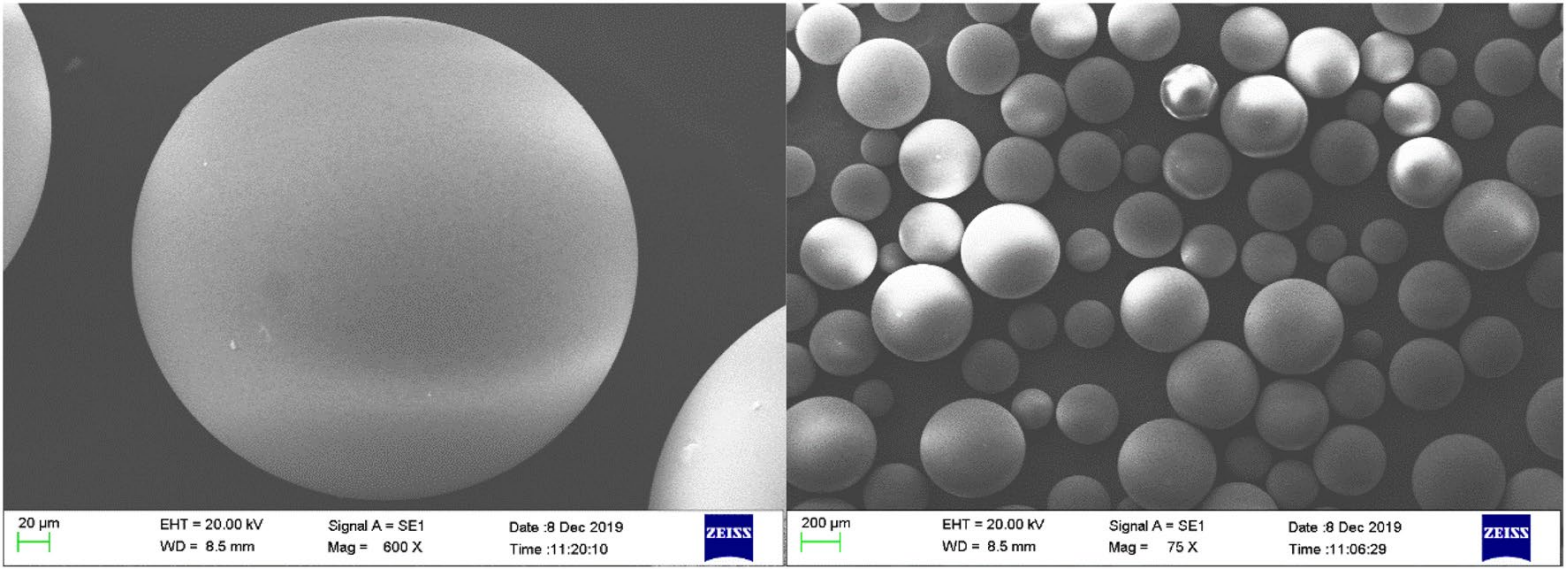
SEM image of Amberlite XAD-7 resin surface washed with distilled water.

**Figure 6:**
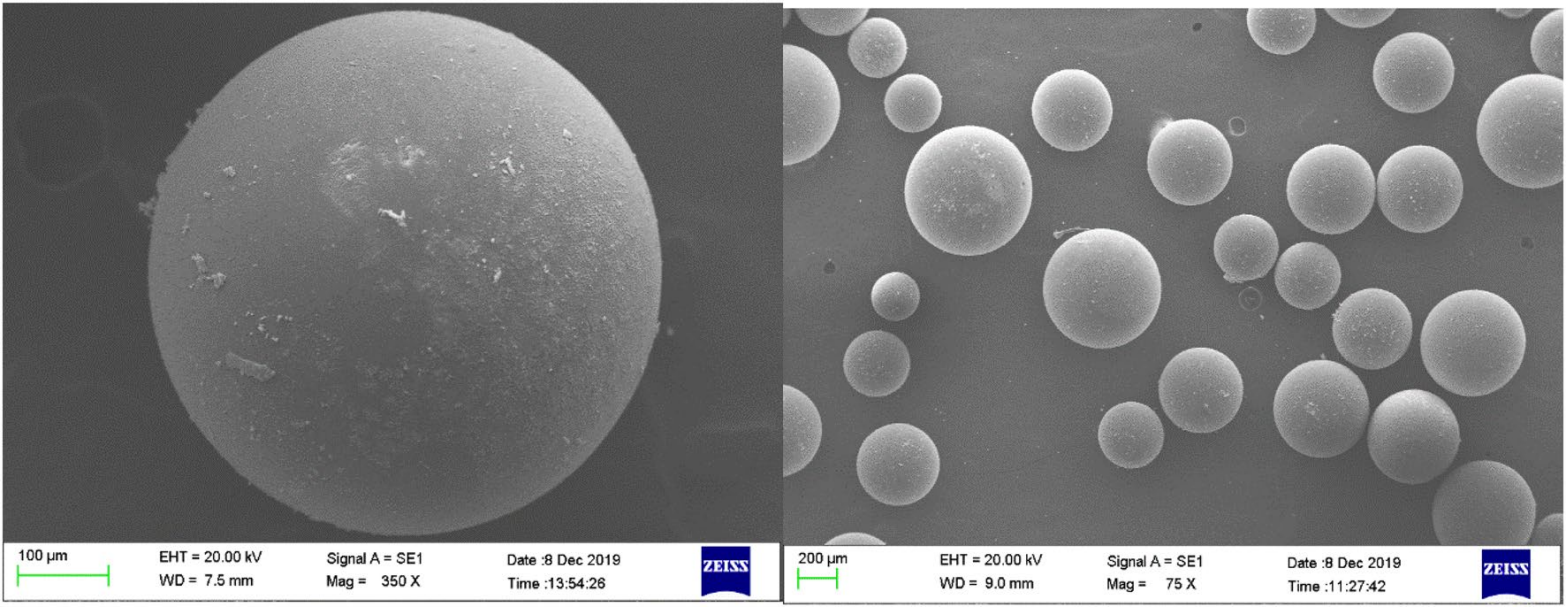
SEM image of Amberlite XAD-7 resin surface after impregnation with DEHP.

SEM images of the Amberlite XAD-7 resin before and after being impregnated with DEHPA are shown in Figs. 5 and 6, respectively. The figures provide the morphologies of the XAD-7 resin and XAD7-DEHPA. The XAD-7 resin exhibited a spherical shape and its high magnification image shows rich pores that can afford abundant space for impregnating extractant reagent DEHPA. By comparing Figs. 5 and 6, changes in morphology can be seen. As can be seen from Fig. 6, the outer surface of the resin has become more opaque after the impregnation process. It is noted that the surface of the XAD7-DEHPA sample (Fig. 6) was similarly constant which implied that the main structure of XAD-7 was retained after the impregnation with DEHPA. It enables XAD7-DEHPA to be a suitable material for ion adsorption in aqueous solutions.

**Figure 7:**
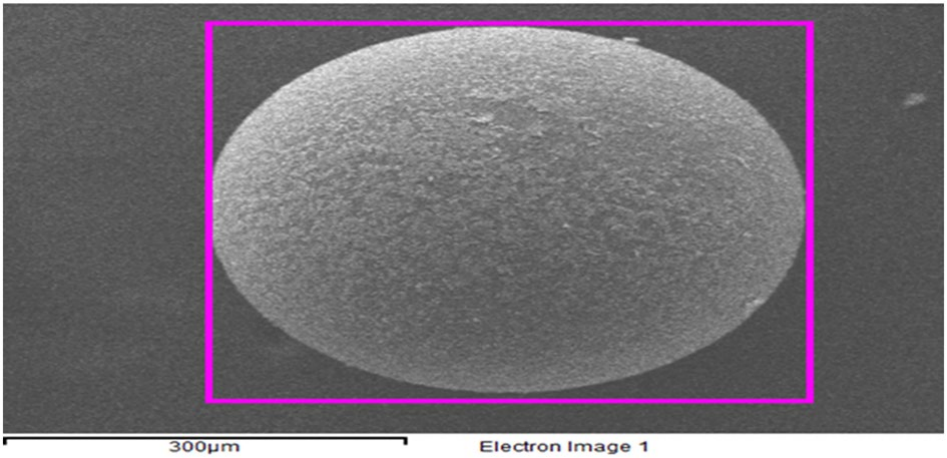
SEM micrographs of the XAD-7-DEHPA after adsorption Ce(IΙI) and La(IΙI).

SEM images of XAD7-DEHPA after adsorption Ce(ΙIΙ) and La(IΙI) are shown in Fig. 7. As can be seen from the figure, the adsorption Ce(ΙIΙ) and La(IΙI) on Amberlite XAD-DEHPA resin surface change the morphology of the beads. In addition, after adsorption, the resin surface became uneven and rough, which could be ascribed to the resin surface being surrounded by La and Ce ions.

**Figure 8:**
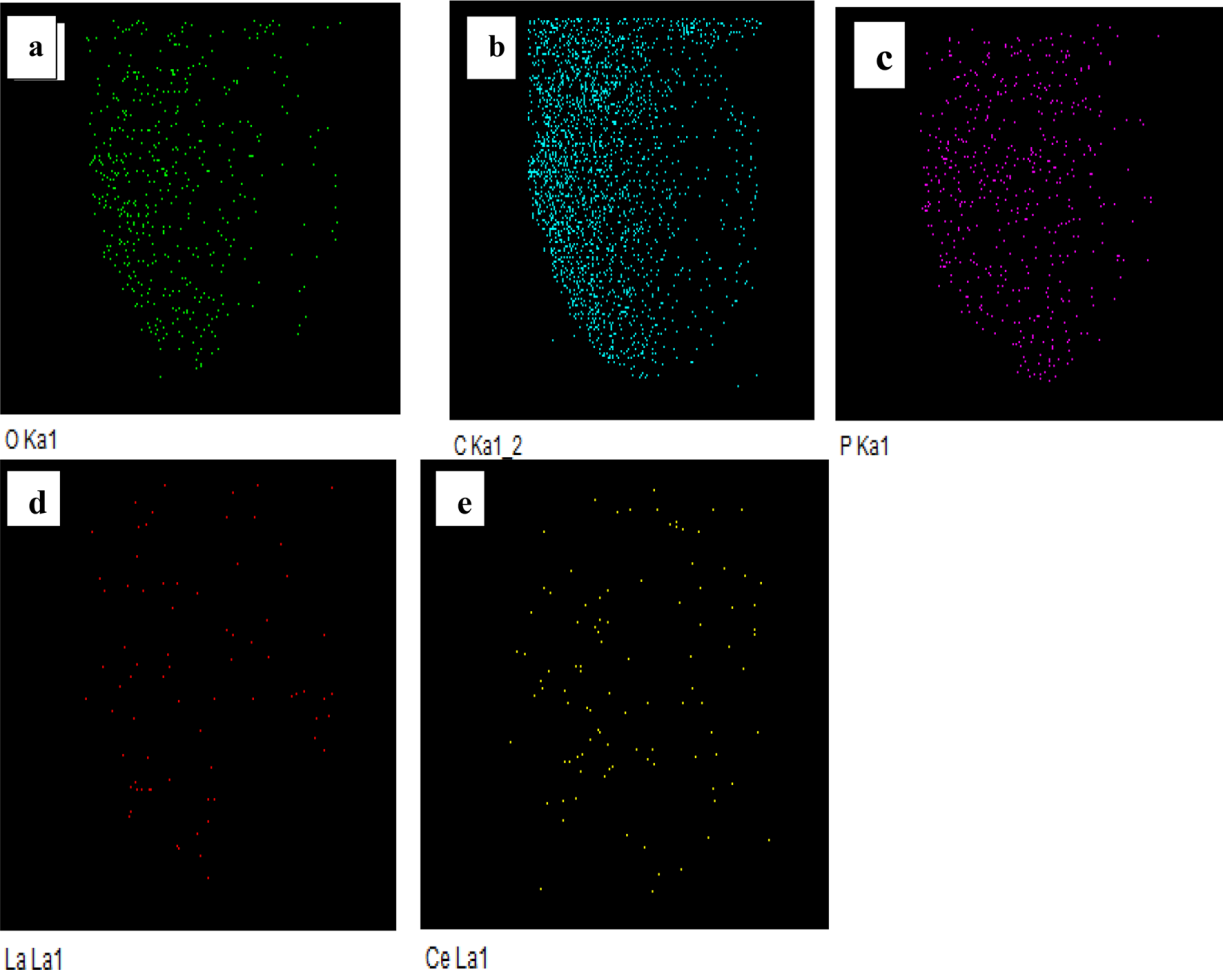
(**a**–**e**) Elemental mapping of O, C, P, Ce(ΙIΙ) and La(IΙI) elements in the XAD7-DEHPA after adsorption of Ce(ΙIΙ) and La(IΙI).

Elemental mapping can a visual impression of the distribution of elements on the XAD-DEHPA resin. Figure 8a–e shows a series of maps acquired. The results demonstrate that there is a distribution of C, O, P, Ce and La element species after the adsorption process on the XAD 7-DEHPA resin.

**Figure 9:**
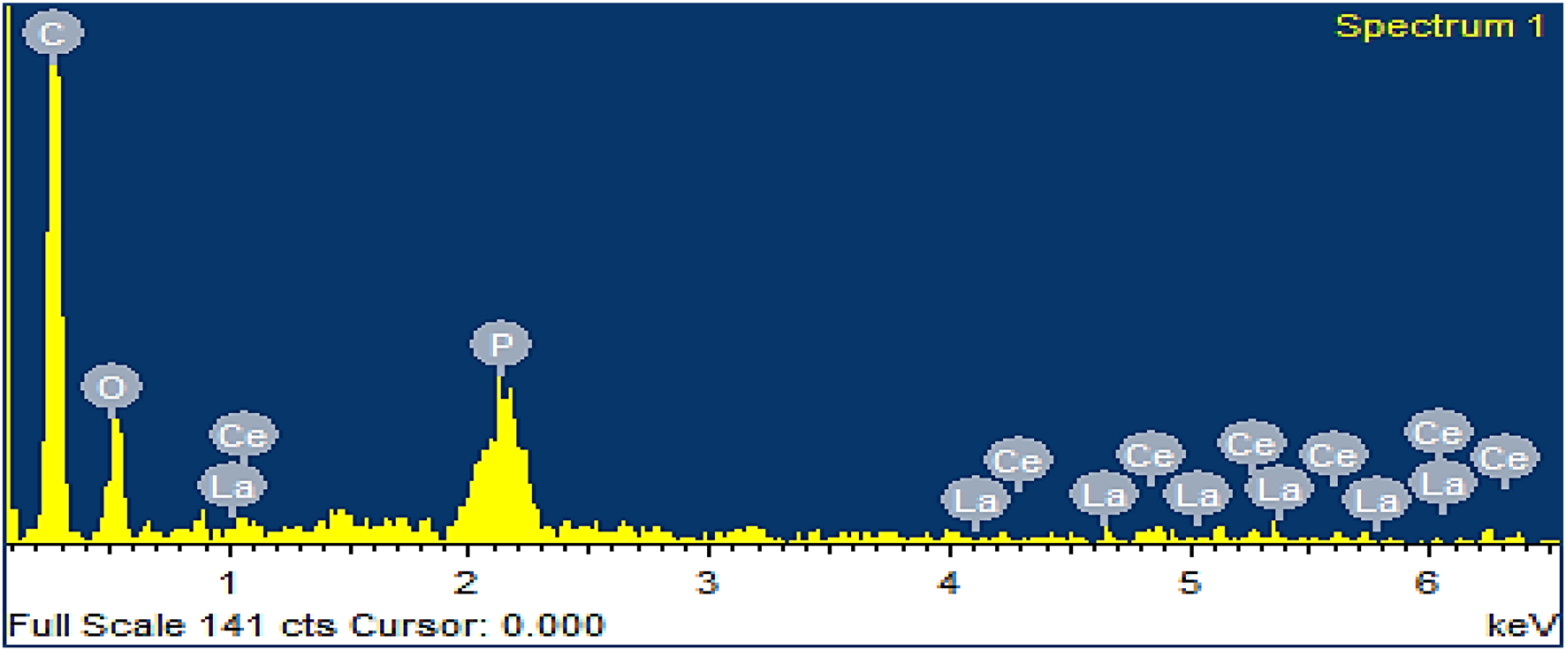
EDX spectrum of XAD7–DEHPA after adsorption of Ce(ΙIΙ) and La(IΙI).

Besides, Fig. 9, indicates the EDX spectrum XAD 7-DEHPA after the adsorption process. The existence of P, O, and C in the spectrum strongly confirms the successful impregnation process using DEHPA, also the presence of the characteristic peak of La(ΙIΙ) and Ce(IΙI), which also proves the adsorption of ions on the XAD7- DEHPA resin.

#### BET surface area

The BET analytical technique is employed to evaluate the specific surface area and pore volume. The surface area and pore size distributions of XAD-7 and XAD7-DEHPA were analyzed by N_2_ adsorption–desorption isotherms are shown in Table 1. The BET surface area was found to be 450 and 58.44 (m^2^/g), also the measured Barrett-Joyner-Halenda (BJH) average pore volume were 1.14 and 0.49 (cm^3^ g^−1^) for XAD-7 and XAD7-DEHPA, respectively. According to the results, after the impregnation process, BET surface area and BJH pore volume of the Amberlie XAD-7 resin decreased due to the filling of its pores by the DEHPA extractant. Therefore, increasing the pore diameter of the XAD-7 resin after impregnation from 85.63 to 137.08 A° can be an explanation for filling the internal cavities of the particles.

**Table 1:** Results of BET analysis for XAD-7 resin after impregnation with DEHPA.

Resin	BET Surface area (m^2^/g)	BJH Adsorption average pore volume(cm^3^ g^−1^)	Adsorption pore diameter (Å)
Amberlite XAD-7	450	1.14	85.63
Amberlite XAD7-DEHPA	58.44	0.49	137.08

### Preliminary Investigation of the Impregnation of Resin

In order to evaluate the effect of the impregnation process, several initial adsorption tests were performed using both XAD-7 and XAD7-DEHPA resins under the same conditions (as 200 mg/L, PH = 5 and temperature of 25 °C with 0.5 g of both types of XAD-7 and XAD7-DEHPA resins for 24 h). The results are shown in Table 2. The results confirm that the adsorption of Ce(IIΙ) and La(IΙI) ions on the resin increased after the impregnation process.

**Table 2:** Evaluation of the role of DEHPA extractant on the adsorption process.

Resin	%AD Ce(IΙI)	%AD La(ΙIΙ)
Amberlite XAD-7	20.68	11.31
Amberlite XAD-7 + DEHPA	94.83	60.91

### Modeling and statistical analysis

In the present research, to determine the main effects and interaction of the three independent parameters, solution pH (A), temperature (B) and dosage of resin (C), on the adsorption of Ce(IIΙ) and La(IΙI) by XAD7-DEHPA resin, a CCD was used in RSM. To study the parameters, 20 experiments with 6 central points were designed and performed. The factors and levels used are listed in Table 3.

**Table 3:** Range of design variables in the central composite design ($\alpha$ = axial points).

Variable	Units	Symbols	Levels				
			$-\alpha=-2$	− 1	0	+ 1	$+\alpha=+2$
pH		A	2	3	4	5	6
Temperature	°C	B	15	25	35	45	55
Dosage resin	g	C	0.05	0.2	0.35	0.5	0.65

The experiments were performed at an initial concentration of 200 mg L^−1^ of a solution of Ce(IIΙ) and La(IΙI) ions with a volume of 20 ml of a solution of metal ions for a time of 180 min. The values of experimental variables and responses for the percentage of cerium and lanthanum adsorption are presented in Table 4.

**Table 4:** Central composite design experiments and experimental results with predicted values.

Levels of actual variables				Ce(ΙΙΙ)		La(ΙΙΙ)	
Run	A	B	C	Experimental	Predicted	Experimental	Predicted
1	5	25	0.5	94.83	91.50	60.90	61.01
2	5	45	0.2	41.61	40.43	27.34	27.15
3	3	45	0.2	31.87	30.84	17.80	17.93
4	4	35	0.35	62.28	62.88	39.24	39.32
5	5	25	0.2	39.69	38.46	25.47	24.99
6	4	15	0.35	60.32	61.94	37.15	37.14
7	4	35	0.35	62.23	62.88	39.15	39.32
8	5	45	0.5	98.46	94.99	64.29	63.58
9	4	35	0.35	62.18	62.94	39.14	39.32
10	3	45	0.5	86.79	84.45	52.91	53.76
11	2	35	0.35	52.08	53.48	29.12	28.84
12	4	35	0.05	9.40	9.79	6.7	6.74
13	4	55	0.35	64.14	66.38	41.45	41.71
14	4	35	0.35	62.23	62.88	39.22	39.32
15	3	25	0.2	29.88	29.35	15.72	15.92
16	4	35	0.65	99.75	116.87	72.33	72.49
17	6	35	0.35	72.37	74.87	49.11	49.71
18	4	35	0.35	62.31	62.88	39.17	39.32
19	3	25	0.5	84.69	82.25	50.99	51.03
20	4	35	0.35	61.98	62.94	39.46	39.32

The predicted responses for the percentage of Ce(IIΙ) and La(IΙI) adsorption by XAD7-DEHPA were calculated by the quadratic model. The equations proposed for Ce(IIΙ) and La(IΙI) are obtained by the software as follows:

5 $$\begin{aligned} {\text{Sqrt }}\left( {\% {\text{AD Ce}}} \right) & = + {7}.{93 } + \, 0.{\text{3351 A}} \\ & \quad + \, 0.0{\text{694 B }} + { 1}.{\text{76 C }} + \, 0.00{\text{53 AB}} \\ & \quad - \, 0.0{\text{621 AC }} - 0.00{\text{38 BC }} \\ & \quad + \, 0.0{\text{132 A}}^{{2}} + \, 0.0{\text{197 B}}^{{2}} - 0.{32}0{\text{4 C}}^{{2}} \\ \end{aligned}$$

6 $$\begin{aligned} {\text{Sqrt }}\left( {\% {\text{AD La}}} \right) & = \, + {6}.{27} + \, 0.{42}0{\text{1 A}} + 0.0{\text{918 B}} \\ & \quad + {1}.{\text{48 C }} + \, 0.00{\text{12 AB }} - 0.0{\text{855AC }} \\ & \quad - 0.0{\text{112 BC }} - 0.0{\text{149 A}}^{{2}} \\ & \quad + \, 0.00{\text{12 B}}^{{2}} - 0.{\text{1789 C}}^{{2}} \\ \end{aligned}$$

While Y represents the percentage of adsorption, in the above equations A (pH), B (temperature) and C (dosage of resin) are independent single variables and AB (pH-temperature interaction), AC (pH-dosage of resin interaction) and BC (temperature-dosage of resin interaction) are reciprocal variables. The quadratic sentences of the equation include A^2^ (pH square effect), B^2^ (temperature Square effect) and C^2^ (dosage of resin Square effect).

The coefficients of parameters A, B, and C and the interaction between parameters AB, AC, BC, A^2^, B^2^ and C^2^ indicate the importance of each of these variables and their interaction with the value of the response (Y). According to the coefficients, three linear effects of factors C (dosage of resin), A (pH) and B (temperature) have the greatest effect on the responses, respectively.

**Figure 10:**
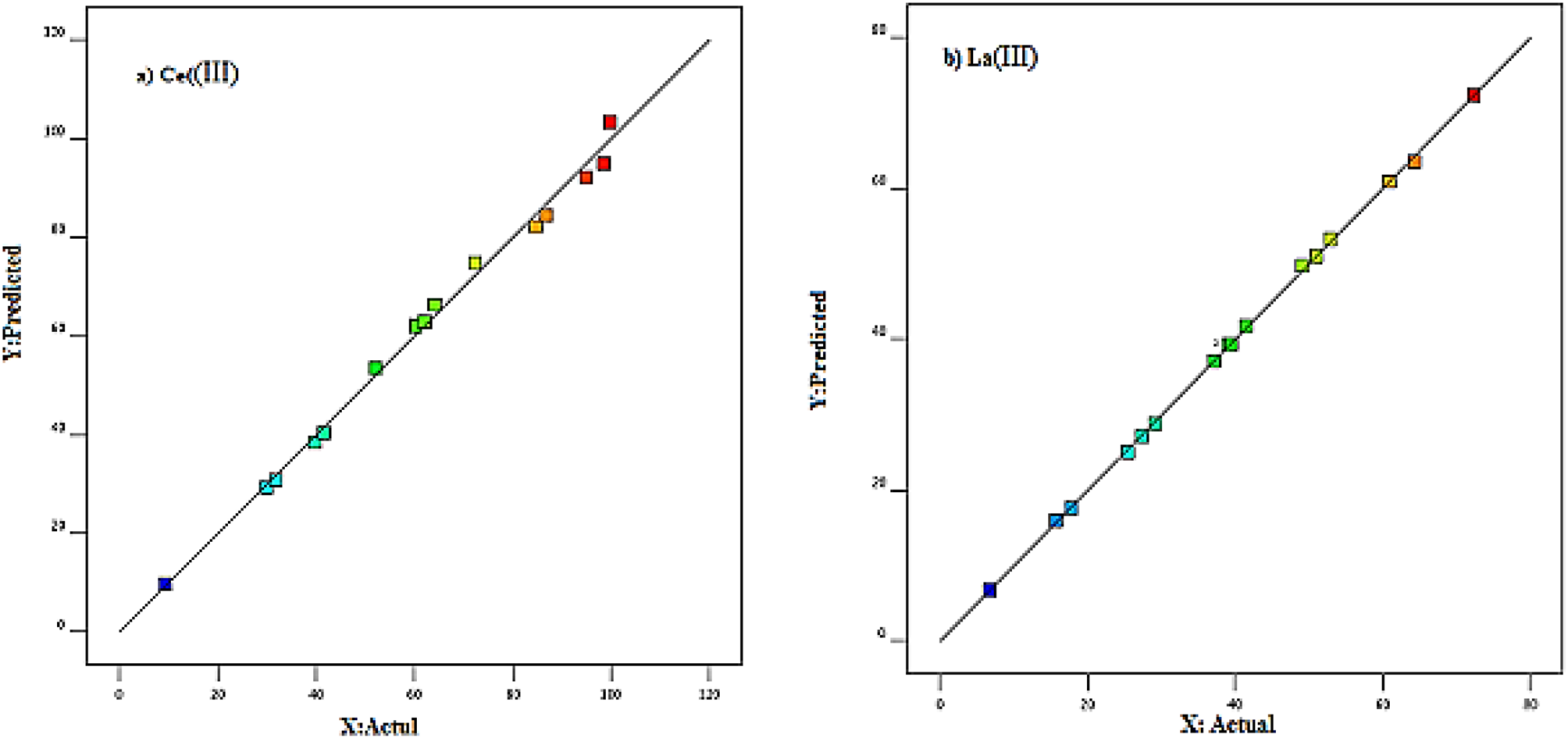
Plots of correlation between actual and predicted values for the percentage of adsorption of Ce(III) and La(ΙIΙ) ions using XAD7-DEHPA.

In discussing model adequacy, Fig. 10 shows the deviation of the experimental data from the predicted data, these figures show that the data are around the 45° line with good dispersion. Given the proximity of the experimental data to the line drawn, it can be concluded that the quadratic equation presented well describes the adsorption process of cerium and lanthanum by the XAD7-DEHPA resin.

Analysis of variance allows the evaluation of the fit of the model with the obtained statistical results. Analysis of variance performed to find the effect of parameters on the percentage of Ce(IIΙ) and La(IΙI) ions adsorption by XAD7-DEHPA resin is shown in Table 5.

**Table 5:** Analysis of variance for the response surface quadratic models.

	Sum of squares	Degree of freedom	Mean square	f	p
Ce(ΙIΙ)					
Model	54.37	9	6.04	274.53	< 0.0001^(s)^
Residual error	0.2200	10	0.0220		
Lack-of-fit	0.2198	5	0.0440	786.11	< 0.0001^(s)^
Pure error	0.0003	5	0.0001		
Total	54.59	19			
R^2^	0.9960				
Adjusted R^2^	0.9923				
La(IΙI)					
Model	38.71	9	4.30	4169.21	< 0.0001^(s)^
Residual error	0.0103	10	0.0010		
Lack-of-fit	0.0097	5	0.0019	15.06	0.0049^(n.s)^
Pure error	0.0006	5	0.0001		
Total	38.72	19			
R^2^	0.9997				
Adjusted R^2^	0.9995				

The Mean squares are the result of dividing the total squares by the degree of freedom. The ratio of the mean squares of the results to the mean squares of the errors represents the F-value index, which indicates the effect of each variable as well as their interaction effects on the response. Its large size indicates the high impact of the variable on the response. The p-value index is used to determine the significant threshold of the variables. Due to the 95% confidence interval, the significance of the variables per p-value is 0.05. The F-value 274.53 and 4169.21for Ce(III) and La(III) along with low probability values (P < 0.0001) demonstrating that the proposed models are significant^36,37^.

R^2^(coefficient of determination) is the model determination coefficient, which indicates the extent to which the results of the proposed model are consistent with the data obtained from the results. Increasing the number of independent variables as well as increasing the degree of freedom increases the coefficient R^2^, therefore, for reference, another coefficient is defined as the adjusted coefficient R^2^_Adj_ (Adjusted R^2^). The proximity of this coefficient to the number one indicates the high accuracy of the model, so considering the value of R^2^_Adj_, which is equal to 0.9923 and 0.9997 for Ce(IIΙ) and La(IΙI), respectively, indicates that the proposed model has high accuracy and the model can establish a good correlation between variables and responses^38^.

Adequate precision (Adeq precision) determines the accuracy and precision of the model and should have a value of more than 4. Given values greater than 4 Adeq precision (for Ce(IIΙ) (67.1130) and La(IΙI) (259.8866)), the obtained models have good accuracy. The distribution of data relative to the mean is shown using the coefficient of variation(C.V %) and the accuracy of the model is expressed in values less than 10. The results in Table 6 confirm this^39^.

**Table 6:** Regression coefficients and their significances in the quadratic polynomial regression equations for percentage extraction of Ce(ΙIΙ)and percentage extraction of La(IΙI).

	Sum of squares	Degree of freedom	Mean square	f	p
Ce(ΙIΙ)					
A	1.80	1	1.80	81.67	< 0.0001
B	0.07	1	0.07	3.51	0.09
C	49.55	1	49.55	2252.08	< 0.0001
AB	0.0002	1	0.0002	0.01	0.92
AC	0.03	1	0.03	1.40	0.26
BC	0.0001	1	0.0001	0.0054	0.94
A^2^	0.0044	1	0.0044	0.2	0.66
B^2^	0.0097	1	0.0097	0.44	0.52
C^2^	2.58	1	2.58	117.32	< 0.0001
La(IΙI)					
A	2.82	1	2.82	2737.25	< 0.0001
B	0.13	1	0.13	130.77	< 0.0001
C	34.84	1	34.84	33,770.51	< 0.0001
AB	0.00001	1	0.00001	0.01	0.92
AC	0.05	1	0.05	56.69	< 0.0001
BC	0.001	1	0.001	0.97	0.34
A^2^	0.0056	1	0.0056	5.41	0.04
B^2^	0.00001	1	0.00001	0.03	0.85
C^2^	0.8	1	0.8	779.66	< 0.0001

### Investigating three-dimensional response surface plot

**Figure 11:**
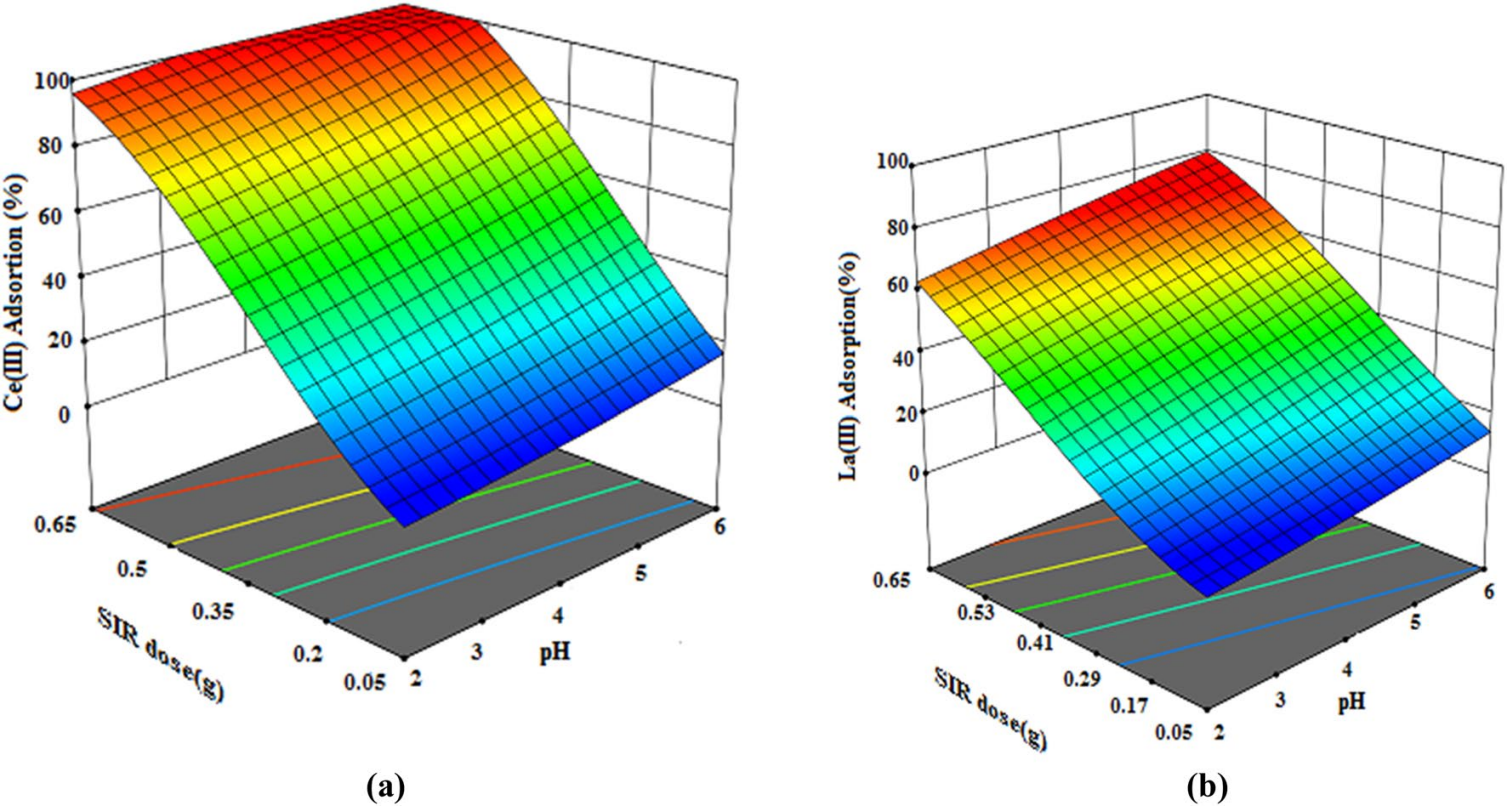
3D response surface plots for the interactive effect of Dosage of resin (g) and pH on the percentage adsorption of (**a**) Ce(III) and (**b**) La(III) by XAD7–DEHPA (initial metal ions concentration 200 mg L^–1^, temperature = 35 °C and time 180 min).

**Figure 12:**
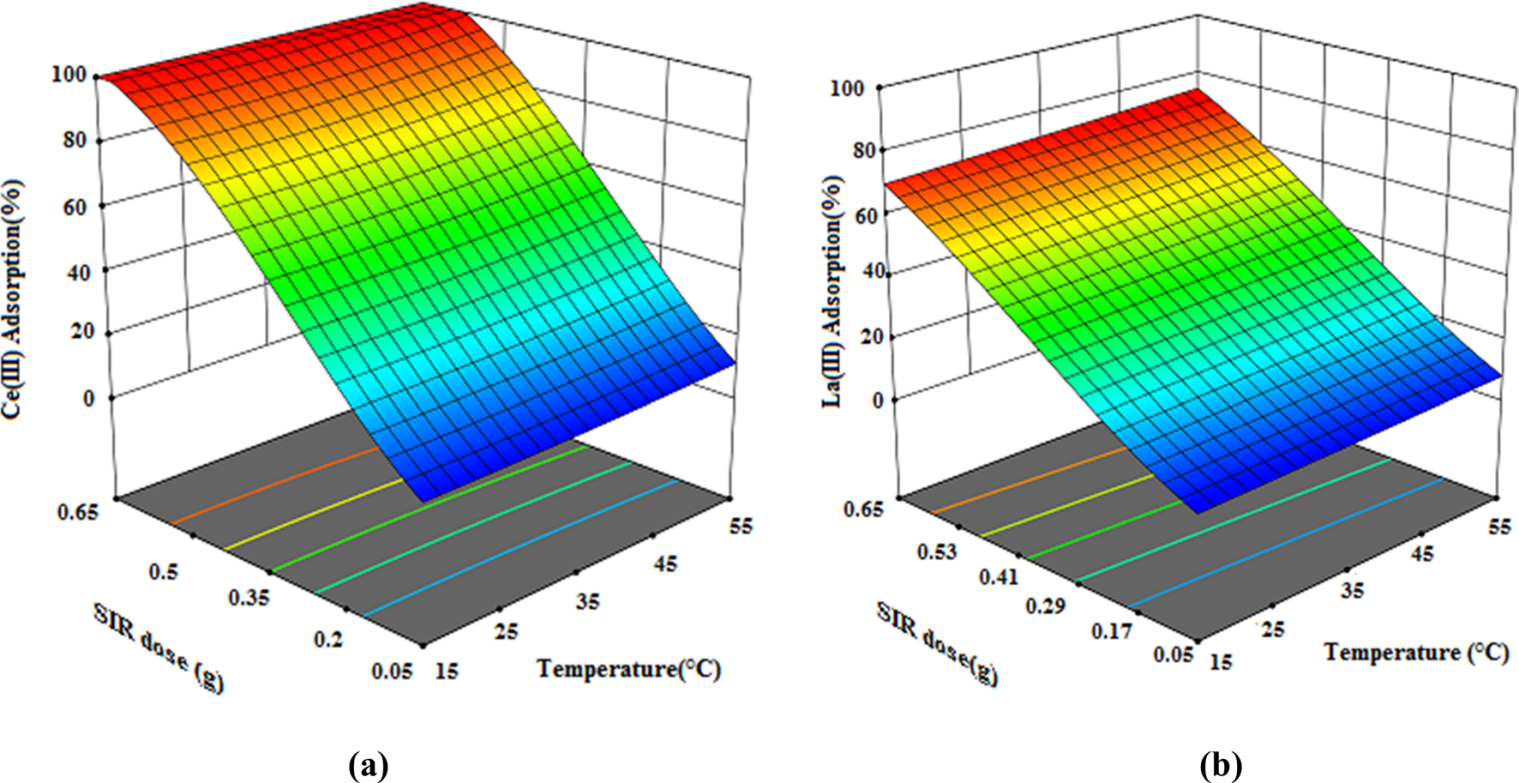
3D response surface plots for the interactive effect of Dosage of resin (g) and temperature (°C) on the percentage adsorption of Ce(III) and La(III) by XAD7–DEHPA (initial metal ions concentration 200 mg L^–1^, pH = 4 and time 180 min).

**Figure 13:**
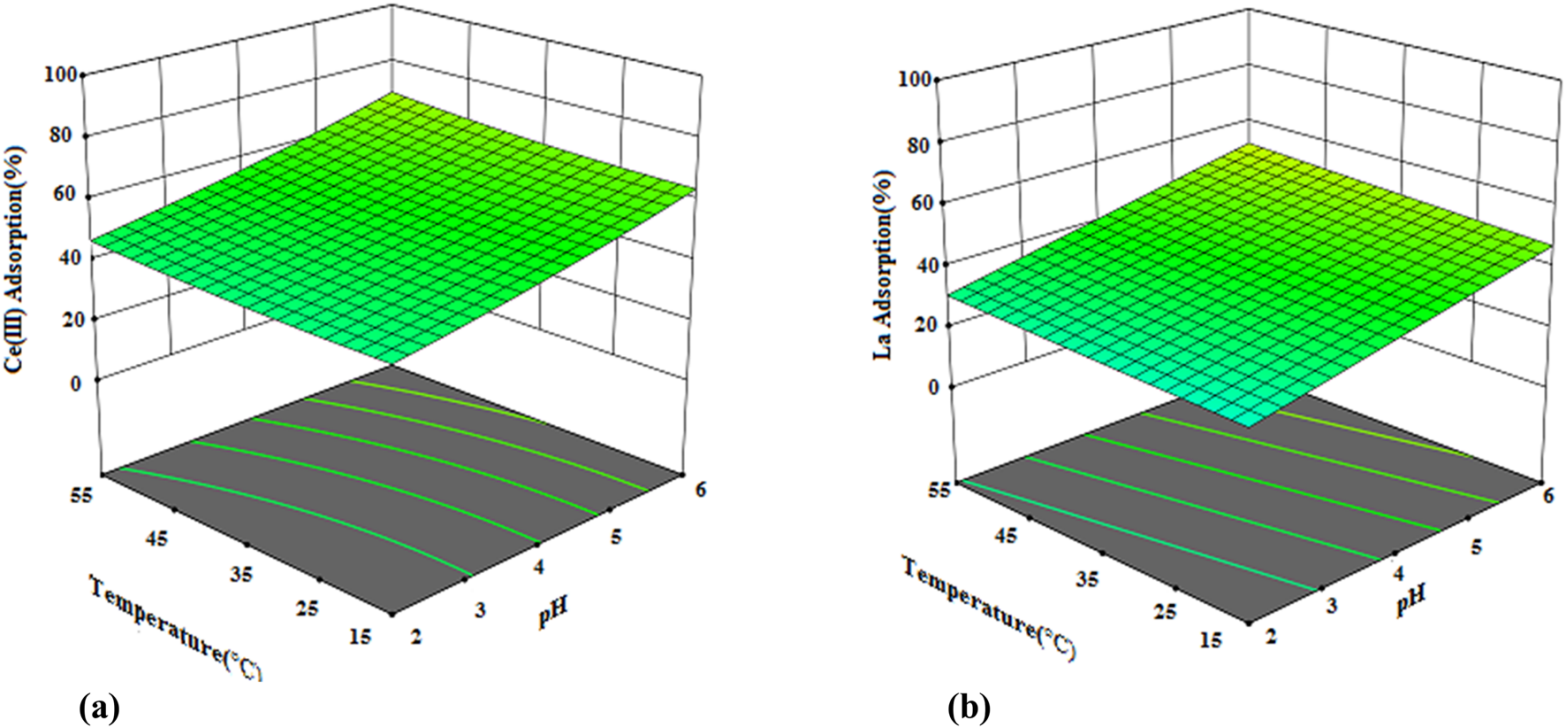
3D response surface plots for the interactive effect of pH and temperature (°C) on the percentage adsorption of Ce(III) and La(III) by XAD7–DEHPA (initial metal ions concentration 200 mg L^–1^, dosage of resin = 0.35 g and time 180 min).

Three-dimensional response surface plots are used to understand the main and interaction effects of two parameters in the adsorption process. Three-dimensional surface plots show the simultaneous effect of two variables on the response while keeping the other variable constant^40,41^. Three-dimensional surface plots of the effect of the interaction of variables (dosage, pH and temperature) on the percentage of Ce(III) and La(III) removal are given in Figs. 11, 12 and 13. Figure 11a,b represent the 3D surface plot for the interaction effect of the Dosage of resin and pH on the Dosage of resin of Ce(III) and La(III) ions. Due to the steep slope of the dosage of resin increasing the dosage of resin from 0.05 g to 0.65 g with increasing pH increases the percentage of removal of Ce(III) and La(III)ions. Increasing the dosage of the resin increases the active binding site for ion adsorption^42^. In addition, the adsorption percentage is more sensitive to the dosage of resin and due to the low slope of the line related to the pH parameter, this parameter has less effect on the dosage of resin than the dosage of resin.

The interaction of temperature and dosage of resin simultaneously on the adsorption of Ce(III) and La(III) ions is seen in Fig. 12a,b. It is obvious that the adsorption percentage of Ce(III) and La(III) increases with increasing temperature and Dosage of resin, but the effect of dosage of resin on the adsorption percentage is much higher than solution temperature. Therefore, the dosage of resin has a greater effect on the adsorption percentage of Ce(III) and La(III) than the other two parameters.

3D surface plots of the interaction effect of pH and temperature on the adsorption percentage of Ce(III) and La(III)are given in Fig. 13a,b. According to Fig. 13a,b although increasing both temperature and pH variables increase the percentage of adsorption, due to the higher slope of the line related to the pH parameter than temperature, the effect of this parameter on the percentage of adsorption is greater. It is also known that increasing the pH from 2 to 6 increases the adsorption of Ce(III) and La(III) because, at low acidic pHs, H^+^ competes with cationic metal ions and the resin bonding sites are occupied by H^+^ and cationic metal ions in solution are less close to the resin due to the repulsive force. At higher pHs, the H^+^ concentration decreases and the resin surface becomes more negatively charged, resulting in an increase in metal ion adsorption^43^.

### Validation of the experimental model

The main purpose of this study was to find the optimal conditions for maximum adsorption of Ce(III) and La(III)using XAD7–DEHPA resin with less time and cost. Experimental response and predicted response by Design-Expert software are reported in Table 7. By comparing the predicted value with the actual value obtained from the experiment, it is concluded that there is a good agreement between the two responses, and the error value is only about 5%, which once again confirms the validity of the quadratic model. The results of this table show the accuracy of the prediction of the experimental points of the model.

**Table 7:** Results of the verification experiments of the reduced model for the maximum percentage of adsorption of Ce(III) and La(III).

Metal ion	pH	Temperature (°C)	Dosage of resin (g)	Predicted %AD	Actual % AD	Error %
Ce(III)	4.76	25	0.577	100	98.64	1.36
La(III)				67.8	65.32	3.65

### Kinetic studies

Investigation of the adsorption process kinetics provides information about the kinetic parameters, the adsorption mechanism, and the step or steps that control the rate of a chemical reaction. For this purpose, the kinetic data of Ce(III) and La(III) adsorption by XAD7–DEHPA resin were modeled using three kinetic models, pseudo-first-order (PFO), pseudo-second-order (PSO) and intra-particle diffusion (IPD) model. Kinetic Studies were performed using the solutions with a volume of 20 mL readied at an initial concentration of 200 mg L^−1^ of Ce(III) and La(III) ions were contacted with 0.35 g of the resin at various times. The linear equations of kinetic models, PFO, PSO and IPD are as follows, respectively^44,45^

7 $$\mathrm{Log }\left(\mathrm{qe }-\mathrm{qt}\right)=\mathrm{ log qe}-\frac{{\mathrm{k}}_{1}\mathrm{ t}}{2.303}$$

8 $$\frac{t}{{q}_{t}}=\frac{1}{{k}_{2}{q}_{e}^{2}}+\frac{1}{{q}_{e}}t$$

9 $${q}_{t}={k}_{id}{t}^{1/2}+c$$

where q_e_ (mg g^−1^) and q_t_ (mg g^−1^) are the equilibrium adsorption capacity and adsorption capacity at time t respectively. k_1_ (min^−1^) is the PFO rate constant, k_2_ (g(mg min)^−1^) is the PSO rate constant, moreover, K_id_ (mg g^−1^ min^−1/2^) and C are the rates constant of IPD and Intra-particle penetration constant respectively.

Adsorption kinetics experiments were designed and performed in the initial concentration of 200 mg L^−1^ solutions of La(III) and Ce(III) ions, the temperature of 25 °C, dosage of resin 0.35 g, pH = 6 in the range time of 5 to 240 min. The kinetic parameters of the quasi-first-order model, the second-order quasi-order model and the intra-particle diffusion model have been calculated using experimental data in terms of time and recorded in Table 8.

**Table 8:** Kinetic Parameters for adsorption of La(III) and Ce(III) by XAD7–DEHPA.

Metal ions	Pseudo-first-order			Pseudo-second-order			Intra partical diffusion		
	qe (mg g^−1^)	K′ (min^−1^)	R^2^	q_e_ (mg g^−1^)	K′_2_ (g mg^−1^ min^−1^)	R^2^	K_id_ (mg g^−1^ min^−1/2^)	C	R^2^
Ce(III)	8.18	0.011	0.99	30.21	0.00005	0.67	0.61	− 1.92	0.97
La(III)	5.19	0.008	0.99	25.12	0.00004	0.53	0.41	− 1.30	0.95

Comparing the coefficient of determination R^2^ (0.99), it is concluded that the process of adsorption of La(III) and Ce(III) by XAD7-DEHPA conforms to the quasi-first-order model which indicates that the chemical reaction is the determining factor of the reaction rate. According to the quasi-first-order model, the number of vacant adsorption sites (adsorption driving force) is proportional to the intensity of occupancy of the adsorption vacancies linearly.

### Adsorption isotherm studies

Adsorption isotherm models are an important factor in the design of adsorption systems, which are used to describe the adsorbent surface properties, provide a perspective on how the adsorption process, and describe experimental data. They are also used to better predict the relationship between the concentration of target species and the adsorbent surface.

In this study, equilibrium data were fitted using Langmuir, Freundlich and Tomkin models. The non-linear equations of the Langmuir, Freundlich and Tomkin adsorption isotherms are as follows, respectively:

10 $${q}_{e}=\frac{{q}_{max}{K}_{L}{c}_{e}}{(1+{K}_{L}{C}_{e})}$$

11 $${q}_{e}={K}_{f}{C}_{e}^\frac{1}{n}$$

12 $${q}_{e}=\frac{RT}{{b}_{T}}\mathrm{ln}{(K}_{T}{C}_{e})$$

According to Eq. (12), q_e_ (mg g^−1^), c_e_ (mg L^−1^) determine the equilibrium concentration of the adsorbent and the amount of equilibrium adsorption, respectively. Maximum monolayer adsorption and adsorption energy are related to Langmuir constant q_m_ (mg g^−1^) and K_L_ (L mg^−1^), respectively. The value of the parameter K_L_ (L mg^−1^) indicates the amount of adsorption between the absorbed and the adsorbent. In Eq. (13), K_f_ (mg g^−1^) shows for Friendlich constant and is associated with the amount of adsorption capacity, and (n) is the dimensionless Friendlich constant that is related to bond strength. The Friendlich parameter n indicates the amount of adsorption force between the adsorbent and the absorbed and has a value between zero and one, and with increasing n the adsorbent forces will be stronger. In Eq. (14), k_T_ and b_T_ are the Temkin isotherm constants, which are the heat of adsorption and the maximum bond energy, respectively. R is the universal constant of gases (8.314 in terms of J mol^−1^ k^−1^) and T is the absolute temperature based on Kelvin^46,47^.

In order to determine the adsorption isotherm of Ce(III) and La(III) by XAD7–DEHPA, a 20 mL solution containing Ce(III) and La(III) ions in different concentrations of 50 to 250 for 180 min at 25 °C was contacted with 0.35 g of resin. According to the data presented in Table 9, the correlation coefficient of the Langmuir model for Ce(III) and La(III) ions is (R^2^ = 0.99), which is higher than the correlation coefficients of the other two models, which Indicates greater agreement of the data with the Langmuir model and single-layer adsorption. Acceptance of this isotherm as an adsorption model for both ions means the uniform distribution of the metal ions extracted on the surface of the resin grains and also the formation of a monolayer coating of ions on the surface of the impregnated resin. The maximum adsorption capacity for Ce(III) and La(III) metals is 8.28 mg g^−1^ and 5.52 mg g^−1^, respectively. In Table 10, the comparison of the maximum absorption capacity of cerium and Lanthanum in the present study with previous researches works results has been shown. According to these results, the values ​​obtained in this study are within the range obtained by other researchers for the absorption of cerium and lanthanum.

**Table 9:** Isotherm constants for adsorption of Ce(III) and La(III) by XAD7–DEHPA.

Isotherm equation	Parameters	Ce(III)	La(III)
Langmuir isotherm	q_m_ (mg g^–1^)	8.28	5.52
	K_L_ (L mg^–1^)	2.04	3.31
	R^2^	0.99	0.99
Freundlich isotherm	K_f_ (mg g^–1^)(L mg^–1^)^–n^	1.86	1.74
	n	5.77	9.84
	R^2^	0.86	0.89
Temkin isotherm	K_T_ (L mg^–1^)	155.37	5681.34
	b_T_ (J mol^–1^)	2683.09	5787.37
	R^2^	0.93	0.91

**Table 10:** Comparison of the adsorption capacity of cerium and lanthanum with different adsorbents.

Adsorbent	Maximum capacity (mg g^−1^)		References
	Ce(III)	La(III)	
calix[4]arene-semicarbazone-merrifield’s peptide resin	1.44	1.87	^48^
Phosphonic acid, Amberlit XAD-4- (o vanillinsemicarbazone)	0.24	0.23	^49^
Polyethylenimine methylene phosphonic acid Merrifield resin	–	1.21	^50^
calix[4]arene-o-vanillinsemicarbazone-merrifield resin	2.82	1.74	^51^
Cyanex- 272- Amberlite XAD-7	11.87	6.32	^52^
Aliquat-336-Amberlite XAD-4	–	0.47	^15^
calix[4]resorcinarene octa-o-methoxy resorcin[4]arene Amberlite XAD-4 polymeric chelating resin	8.64	8.46	^53^
Amberlite-XAD-16-octa carboxymethyl-c-methyl	11.63	10.41	^54^
azocalix[4]pyrrole-Amberlite XAD-2	8.98	11.05	^55^
Corrent work, DEHPA-Amberlite XAD-7	8.28	5.52	

### Sorption mechanism for SIR, Amberlite XAD-7 containing DEHPA

In order to explain the binding mechanism of cerium (III) by various adsorbents or ion exchange materials, it is necessary to understand the solution chemistry of trivalent cerium. The distribution of Ce(III) species is dependent on both the total concentration of Ce(III) and pH of the equilibrium solution. According to Borai and coworkers, cerium exists in four main forms in aqueous solution. The reactions between these species and the reaction are shown in Eqs. (13)–(15)^56^. Knowledge of speciation is vitally important to determine solubility in environmental waters and the interactions of a species with SIR or ion exchange materials. Speciation of cerium (III) in aqueous solution at a specific total concentration of it is highly dependent on the solution pH. This could be interpretated based on the following three equations that demonstrate the successive hydrolysis of Ce(III) in solution:

13 $${\text{Ce}}^{{{3} + }} \left( {{\text{aq}}} \right) \, + {\text{ OH}}^{-} \left( {{\text{aq}}} \right) \, \leftrightarrow {\text{ Ce}}\left( {{\text{OH}}} \right){2}^{ + } \left( {{\text{aq}}} \right)$$

14 $${\text{Ce}}\left( {{\text{OH}}} \right){2}^{ + } \left( {{\text{aq}}} \right) \, + {\text{ OH}}^{-} \left( {{\text{aq}}} \right) \, \leftrightarrow {\text{ Ce}}\left( {{\text{OH}}} \right)^{{{2} + }} \left( {{\text{aq}}} \right)$$

15 $${\text{Ce}}\left( {{\text{OH}}} \right)^{{{2} + }} \left( {{\text{aq}}} \right) \, + {\text{ OH}}^{-} \left( {{\text{aq}}} \right) \, \leftrightarrow {\text{ Ce}}\left( {{\text{OH}}} \right)_{{3}}$$

The obtained results showed that by further addition of OH^–^ (with rising pH) the trivalent Ce ions (Ce^3+^) shift to monovalent ions (Ce(OH)2^+^) and Ce(III) precipitates out of solution after pH 7 as a cloudy white gellike precipitate, which is presumably Ce(OH)_3_(s)^56,57^.

From the comparison experimental results reported in Table 2 to evaluate the effect of the impregnation process, and occurring remarkable enhance in adsorption with XAD7-DEHPA in comparison with XAD-7, can be result that DEHPA reagent is main responsible for adsorption of Ce(IIΙ) and La(IΙI) ions on the adsorbent and Amberlite resin mainly acts as solid support in adsorbent structure.

**Figure 14:**
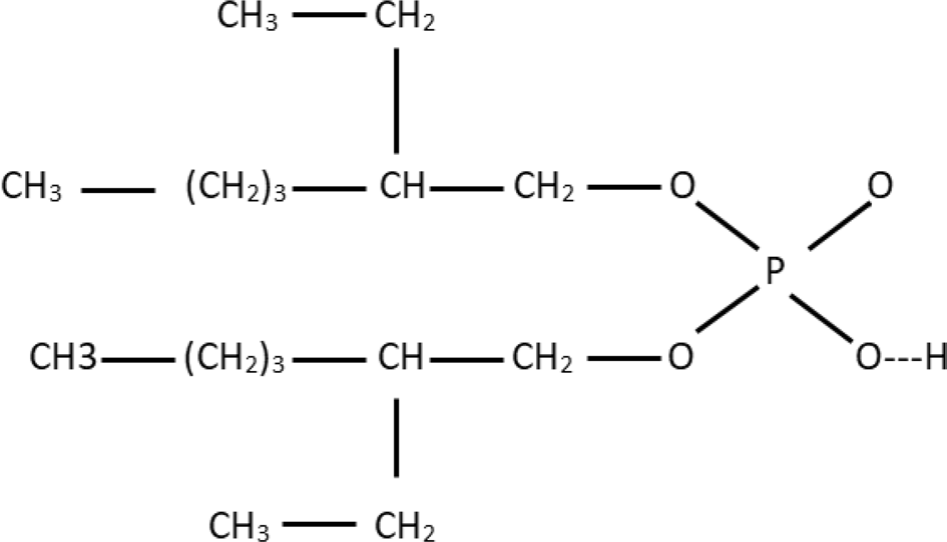
Molecular structure of DEHPA extractant.

The chemical formula for DEHPA is (C_8_H_17_O)_2_PO_2_H and the proposed structure is shown in Fig. 14. DEHPA contains a hydrogen ion in oxygen-hydrogen bound in its structure as shown in Fig. 14. This hydrogen ion could potentially be exchanged for a monovalent Ce(OH)2^+^ ion as it has a single positive charge. The cerium divalent species (Ce(OH)^2+^) could potentially be exchanged for two hydrogen ions as it has two positive charges and so on trivalent Ce^3+^ species could potentially be exchanged for three hydrogen ions. Therefore, monovalent ions sorption, as it requires one DEHPA active site to bond with, will be much easier than divalent and trivalent ions which requires two and three DEHPA active sites to bond with, respectively. From the speciation of cerium (III) ions, at upper pHs, the cerium ions are often present in solution as monovalent ions (Ce(OH)2^+^). According to Fig. 13a,b increasing pH variable increase the percentage of adsorption, which can be attributed to the presence of monovalent cations at high pHs in solution. At lower pHs, the predominant presence of trivalent cations in solution decreases the absorption percentage. Testing for the increasing of H^+^ ions in the aqueous solution after exposure to an SIR could check the proposed mechanism.

At alkaline pHs the sorption efficiency of cerium ions was significantly lower. One potential reason for this is that there is a higher concentration of the non-ionic species (Ce(OH)_3_) at alkaline pHs. As this species carries no charge, the SIR will not be able to remove it through ion exchange.

These evidences show that the main adsorption mechanism in the Ce(IIΙ) ions removal by Amberlite XAD7-DEHPA resin is ion exchange and the extractant DEHPA acts as a cation exchanger agent. Similar results have been obtained from other studies for the metallic ions, lanthanum (III) and cerium (III), recovery using a mixture of 2-ethylhexylphosphonicmono-2-ethylhexyl ester and di-2-ethylhexyl phosphoric acid (DEHPA)^58^. Moreover, the coordination effect between Ce and P=O ligands in DEHPA was verified using the ^31^P NMR spectra of the organo-Ce complexes by Zhang et al.^58^.

Considering the similar results of pH effect on lanthanum adsorption, a similar mechanism can be true for lanthanum and similar inferences can be made as the results of Zhang et al. work also confirm this^58^.

### Desorption and regeneration studies

The literature review indicates that SIRs are efficiently employed for the extraction of metal ions with well stability during several adsorption–desorption cycles^52^. In present work also, the used Amberlite XAD-7 resin impregnated via D2EHPA extractant was recovered 3 times and showed high stability during 3 adsorption–desorption cycles.

**Figure 15:**
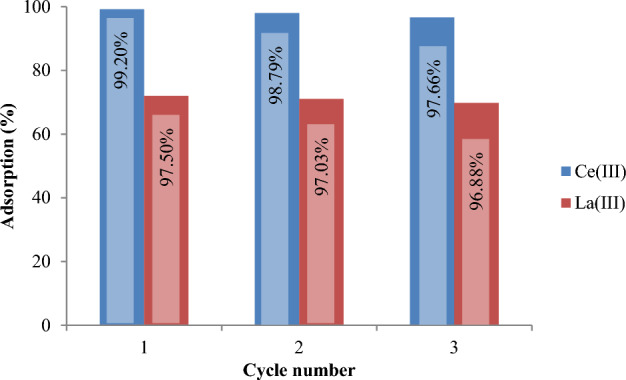
Adsorption–desorption efficiency of XAD7–DEHPA resin in 3 consecutive cycles using HCL 0.1 mol L^−1^.

**Figure 16:**
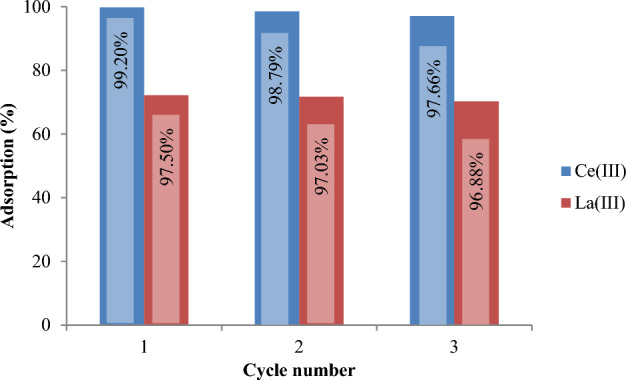
Adsorption–desorption efficiency of XAD7–DEHPA resin in 3 consecutive cycles using HCl 2 mol L^−1^.

The results obtained from three processes of adsorption and desorption of Ce(III) and La(III) by XAD7–DEHPA can be seen in Figs. 15 and 16, respectively. These results show that the resins used for 3 cycles have a recycling percentage higher than 95% and can be used in several adsorption and desorption cycles without a significant reduction in adsorption. In addition, with increasing the concentration of nitric acid from 0.1 to 2 mol L^−1^, the desorption of Ce(III) and La(III) ions increases slightly.

## Conclusions

In this study, the adsorption process of cerium and lanthanum ions from aqueous solutions was investigated using Amberlite XAD-7 resin in a batch system. The structure and shape of the resin before and after impregnation by the extraction were well determined by FT-IR, BET, SEM-EDX analysis. The RSM was used to optimize the variables affecting the adsorption process of Ce(III) and La(III). The obtained results showed that the optimal values of the effective variables are equal to the dosage of resin 0.577 g, pH = 4.76 and temperature of 25 °C in order to obtain the maximum removal of 98.64% Ce(III) and 65.32 La(III) by XAD-7-DEHPA. Langmuir, Freundlich, and Temkin isotherm models were used to describe the equilibrium data, and the best fit was performed with the Langmuir model (R^2^_(Ce)_ = 0.999, R^2^_(La)_ = 0.998). The maximum adsorption capacity of resin for cerium and lanthanum was 11.17 and 6.48 mg g^−1^, respectively. The kinetic study was performed using PFO, the PSO and IPD models. According to the results, kinetic data for Ce and La adsorption were fitted well to a pseudo-second-order rate equation (R^2^_(Ce)_ = 0.966, R^2^_(La)_ = 0.985). Adsorption–desorption studies during three cycles of use and recycling showed that this impregnated resin is economical and very effective in removing the two elements Ce(III) and La(III).

## Data Availability

The datasets used and/or analyzed during the current study are available from the corresponding author on reasonable request.
